# RETRACTION: Suppression of lncRNA MALAT1 Reduces LPS‐ or IL‐17A‐Induced Inflammatory Response in Human Middle Ear Epithelial Cells via the NF‐*κ*B Signaling Pathway

**DOI:** 10.1155/bmri/9840127

**Published:** 2026-06-02

**Authors:** BioMed Research International

RETRACTION: X. Yang, Q. Zhang, H. Lu, C. Wang, and L. Xia, “Suppression of lncRNA MALAT1 Reduces LPS‐ or IL‐17A‐Induced Inflammatory Response in Human Middle Ear Epithelial Cells via the NF‐*κ*B Signaling Pathway,” BioMed Research International 2021, no. 1 (2021): 8844119, 10.1155/2021/8844119.

The above article, published online on 7 January 2021 in Wiley Online Library (wileyonlinelibrary.com), has been retracted by agreement between the journal Editor‐in‐Chief and John Wiley & Sons Ltd. The retraction has been agreed due to figure concerns identified within the western blots presented in Figures 1 and 3.

More specifically:

• In Figure 1E, the *β*‐actin appears to be duplicated with the GAPDH panel in Figure 1E of [1].
• In Figure 3D, the Nucleus p65 panel, the IL‐17A and IL‐17A + sh‐NC bands appear to be duplicated with the E‐cadherin panel in Figure 5F of [2]. There also appears to be an undeclared splice in this figure.

As a result of the investigation, the data and conclusions of this article are considered unreliable.

The authors did not respond when notified of the retraction.
